# Coexistence of Cardiovascular Risk Factors and Blood Renalase Concentration

**DOI:** 10.3390/ijms242316666

**Published:** 2023-11-23

**Authors:** Aleksandra Żórawik, Wojciech Hajdusianek, Iwona Markiewicz-Górka, Aleksandra Jaremków, Krystyna Pawlas, Helena Martynowicz, Grzegorz Mazur, Rafał Poręba, Paweł Gać

**Affiliations:** 1 Department of Population Health, Division of Environmental Health and Occupational Medicine, Wroclaw Medical University, Mikulicza-Radeckiego 7, PL 50-368 Wroclaw, Poland; 2 Department of Internal Medicine, Occupational Diseases, Hypertension and Clinical Oncology, Wroclaw Medical University, Borowska 213, PL 50-556 Wroclaw, Poland

**Keywords:** renalase, cardiovascular health, CVRF

## Abstract

Cardiovascular diseases (CVDs) are one of the biggest health challenges facing health systems around the world. There are certain risk factors (CVRFs) that contribute to CVD. Risk factors associated with lifestyle such as tobacco consumption are particularly essential. Renalase is a recently discovered flavoprotein that may be involved in the progression of cardiometabolic diseases. The aim of the study was to investigate the relation between CVRFs and blood renalase concentration (BRC). The study group consisted of 96 people (51% women) who were hospitalized in the internal medicine department. CVRFs were measured using the AHA Life 7 scale. The E3109Hu ELISA kit was used to assess BRC. We found higher BRC in groups with a lower number of CVRFs (*p* < 0.05). We found a negative correlation between BRC and the number of CVRFs (r = −0.41). With the regression analysis, obesity, smoking, and a lack of physical activity (LoPE) were independently associated with lower blood renalase concentration. ROC analysis indicated the highest accuracy of BRC < 38.98 ng/mL in patients with ≥5 CVRFs. In conclusion, patients with a higher number of CVRFs had lower BRCs. The CVRFs particularly associated with a lower BRC were obesity, smoking, and LoPE.

## 1. Introduction

Cardiovascular disease (CVD) is one of the biggest health challenges facing health systems around the world. According to the World Health Statistics 2023, non-communicable diseases (NCDs) continued to cause the highest disease burden worldwide: the impact of NCDs grew from causing 61% of global deaths (31 million) in 2000 to 74% (41 million) in 2019. In total, the four major NCDs caused the deaths of about 33.3 million people in 2019 (this represents an increase of 28% in 2000). Of the four major non-communicable diseases (cardiovascular disease, cancer, chronic respiratory disease, diabetes), cardiovascular disease accounts for the largest share of global mortality (17.9 million) [1,2,3]. However, the increase in the absolute number of deaths caused by NCDs was mainly due to population growth and aging and at the individual level, the overall risk of death from NCDs is declining, showing progress over the last twenty years [2,4].

The World Health Organization defines cardiovascular diseases as “a group of disorders of the heart and blood vessels, including coronary heart disease, cerebrovascular disease, peripheral arterial disease, rheumatic heart disease, congenital heart disease and deep vein thrombosis and pulmonary embolism” [3].

According to current knowledge, the onset of non-communicable diseases is the resultant influence of behavioral, environmental, physiological, and genetic factors. Some of these risk factors are modifiable, which include harmful alcohol consumption, smoking, physical inactivity, and an unhealthy diet. Metabolic risk factors include elevated blood pressure, overweight and obesity, hyperglycemia (high blood glucose levels), and hyperlipidemia (high blood fat levels). What is more, prevention of non-communicable diseases can be supported by improved environmental factors—including proper water hygiene, improving air quality, safe use of chemicals, ensuring safe and hygienic working conditions, protection against radiation, or stopping/slowing down climate change and environmental degradation [2].

In the context of cardiovascular health and CVD prevention, lifestyle is key to reducing the incidence of these diseases [5]. The most strongly documented cardiovascular disease risk factors (CVRFs) include tobacco use, arterial hypertension (AH), hypercholesterolemia, hypertriglyceridemia, type 2 diabetes mellitus (DM), and excessive body weight [6,7,8,9]. Therefore, cardiovascular diseases can be prevented by avoiding modifiable behavioral risk factors such as smoking, an unhealthy diet, alcohol abuse, excessive body weight, and physical activity deficiency [3,10].

At the same time, the presence of all the mentioned risk factors is common, often as a coincidence of many of them. In the context of the most serious CVRFs (smoking cigarettes, AH, hypercholesterolemia and hypertriglyceridemia, DM, overweight and obesity), the statistics are as follows [7,8,11]: in 2012, 21% of the world’s population aged 15 and over smoked cigarettes (in Poland 26% [12]); in 2014, 22% of the adult population had hypertension (in Poland 33% [12]); in 2008, nearly 40% of adults worldwide were diagnosed with lipoprotein metabolic disorders (in Poland 64% of women and 70% presented abnormally high total cholesterol levels—according to the results of the WOBASZ II study [13,14]); in 2014, 13% of the world’s population was obese and 39% was overweight (in Poland, one in four people were obese [15]); in 2014, almost 9% of adults aged 18 years and older had diabetes [16] (in Poland 7% in 2021) [12,17]. Previous studies indicate that the coexistence of CVRFs may worsen the prognosis of various cardiovascular diseases, including congestive heart failure [18].

Renalase is a relatively recently discovered flavoprotein that remains a subject of scientific interest [19]. Renalase was first described in 2005 as an effort to increase knowledge of renal endocrine function [20,21,22]. Initially, the function of renalase—this novel flavin adenine dinucleotide-dependent (FAD-dependent) amine oxidase—was described as responsible for slowing down the heart and the regulation of blood pressure due to its ability to catabolize circulating catecholamines [20,21,23]. However, further research has challenged this theory [19,24,25,26]. The results of later studies described renalase as acting as α-NAD(P)H oxidase/anomerase and oxidizing α-NAD(P)H to β-NAD(P)+ and H_2_O_2_ [27]. Further analysis has updated these findings, demonstrating that renalase oxidizes two specific substrates, namely 2- and 6-dihydroNAD(P), resulting in the inhibition of primary metabolism dehydrogenase, producing β-NAD(P)+ and H_2_O_2_ [28,29]. Renalase was also linked to factors associated with dysfunction of the endothelium. Recently, renalase was also found to have anti-inflammatory and anti-apoptotic effects as a response to ischemia [30].

In previous studies on the relationship between the risk factors of cardiovascular disease and blood renalase concentration, most data were collected in relation to arterial hypertension [31]. Studies on the relationship between, on the one hand, obesity, lack of physical activity, smoking, diet, and dyslipidemia, and on the other hand, the blood renalase concentration are few, ambiguous, and mainly concern animal models.

The aim of the study was to investigate the relationship between the coexistence of cardiovascular risk factors and blood renalase concentration.

## 2. Results

### 2.1. Basic Demographic and Clinical Parameters

The studied population was composed of 96 participants: 49 (51%) females and 47 (49%) males. The mean age of participants was 48.51 ± 14.73 years and, in particular, 50.64 ± 14.12 years in males and 46.47 ± 15.15 years in females. Among the participants, 33 (34.4%) were obese, 17 (36.2%) males and 13 (26.5%) females. The mean body mass index in the study group was 27.82 ± 4.78 kg/m^2^, with 27.92 ± 3.77 kg/m^2^ for males and 27.71 ± 5.61 kg/m^2^ for females.

Arterial hypertension occurred in 34 patients (35.4%). The mean systolic blood pressure was 136.77 ± 19.65 mmHg and the mean diastolic blood pressure was 87.24 ± 11.17 mmHg. The number of participants with hypercholesterolemia was 42 (43.7%) and the mean total cholesterol was 217.48 ± 39.17 mg/dL. Diabetes mellitus occurred in eight patients (8.3%) The mean fasting glucose concentration was 116.75 ± 41.18 mg/dL. In the study group, 27 (28.1%) of the participants were smokers. The mean cardiovascular risk factor was 2.19 ± 1.42. We found no significant differences between males and females in our study in the parameters described above.

The number of cardiovascular risk factors in the study group was as follows: no CVRFs were found in 13 patients (13.54%), one CVRF in 24 patients (25%), two CVRFs in 22 patients (22.9%), three CVRFs in 17 patients (17.7%), four CVRFs in 11 patients (11.45%), five CVRFs in eight patients (8.33%), and six CVRFs in one patient (1%). There were no participants with seven CVRFs.

The mean blood renalase concentration was 212.89 ± 281.91 ng/mL. The minimum blood renalase concentration was 26.70 ng/mL and the maximum concentration was 790.48 ng/mL.

### 2.2. Comparative Analysis: Cardiovascular Risk Factors and Blood Renalase Concentration

In our study, we analysed blood renalase concentration differences between the groups that differed on each individually analysed cardiovascular risk factor. Among the statistical results, we found that blood renalase concentration was higher in the non-smoking, without obesity, with appropriate physical activity, and without arterial hypertension groups. Normal fasting glucose was also related to the blood renalase concentration. The group with normal fasting glucose was less likely to have blood renalase concentration < the first quartile, see Table 1.

### 2.3. Comparative Analysis: Number of Cardiovascular Risk Factors and Blood Renalase Concentration

We studied the blood renalase concentration in the groups when defined by the number of cardiovascular risk factors. We found a higher blood renalase concentration in the groups with a lower number of CVRFs, see Table 2.

### 2.4. Correlation Analysis

**Figure 1:**
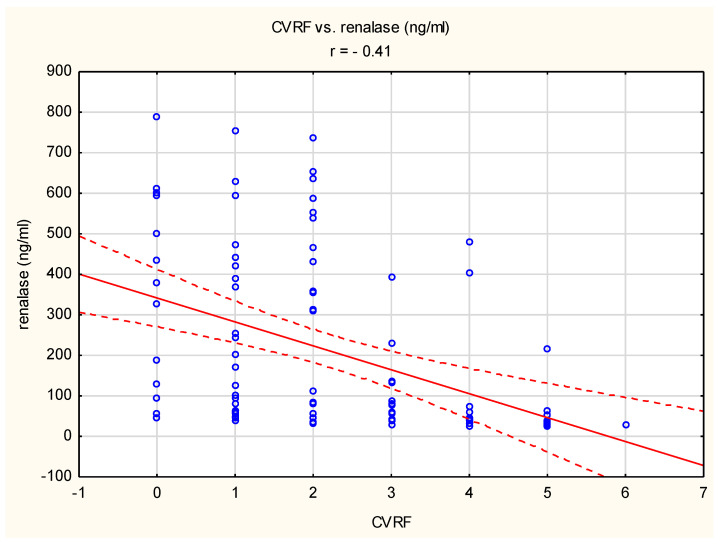
Correlation between the number of cardiovascular risk factors (CVRFs) and the blood renalase concentration. Blue dots indicate individual cases, red line indicates the correlation line, red dashed lines indicate the confidence interval (±95%) of the correlation line.

We found a negative correlation between the number of cardiovascular risk factors and the blood renalase concentration with r = −0.41, see Figure 1.

### 2.5. Regression Analysis

The results of the multivariate regression analysis are summarized in Table 3. We used a progressive stepwise analysis method.

Considering cardiovascular risk factors, the following statistically significant relationship model was obtained: renalase [ng/mL] = 333.195 − 121.748 obesity − 118.115 active smoking − 87.029 lack of physical activity. The presence of obesity, being a current smoker, and a lack of physical activity are independently linked with a higher probability of lower blood renalase concentration.

### 2.6. Prediction Analysis

We analysed blood renalase concentration as a predictor of the number of cardiovascular risk factors. The sensitivity and specificity of the analysis are presented in Table 4. 

**Figure 2:**
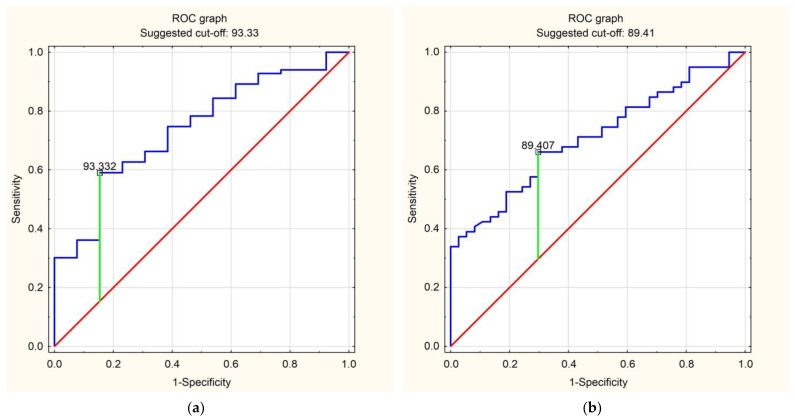

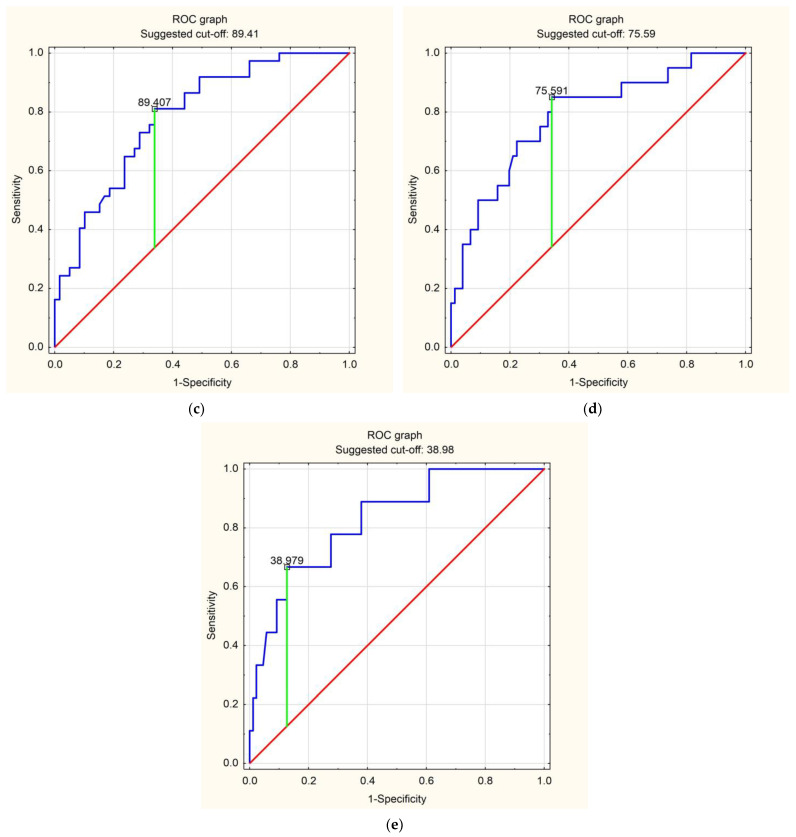
ROC curves predicting the number of CVRFs using blood renalase concentration. The red line denotes the baseline of the analysis, the blue line denotes the prediction line in the analysis, the green line locates the optimal cut-off point in the analysis. (**a**) CVRF ≥ 1 predicted by blood renalase concentration; (**b**) CVRF ≥ 2 predicted by blood renalase concentration; (**c**) CVRF ≥ 3 predicted by blood renalase concentration; (**d**) CVRF ≥ 4 predicted by blood renalase concentration; (**e**) CVRF ≥ 5 predicted by blood renalase concentration.

The conducted receiver operating characteristic (ROC) analysis indicated blood renalase concentration values constituting predictor conditions for the number of CVRFs, being less than 93.33 ng/mL for CVRF ≥ 1, less than 89.41 ng/mL for CVRF ≥ 2, less than 89.41 ng/mL for CVRF ≥ 3, less than 75.59 ng/mL for CVRF ≥ 4, and less than 38.98 ng/mL for CVRF ≥ 5. The highest calculated prediction sensitivity of 0.874 was demonstrated for a blood renalase concentration < 38.98 ng/mL—the predictor of CVRFs ≥ 5. The highest specificity was 0.800 for a blood renalase concentration < 75.59 ng/mL—the predictor of CVRF ≥ 4. Overall, the highest prediction accuracy was 0.844 for a blood renalase concentration < 38.98 ng/mL—the predictor of CVRF ≥ 5. A summary of the ROC analysis is presented in Figure 2.

## 3. Discussion

### 3.1. Most Important Findings

In our study, we observed a negative relationship between the number of cardiovascular disease risk factors and the blood renalase concentration. The occurrence of obesity, being a current smoker, and physical inactivity are independent risk factors for a lower blood renalase concentration. Lower blood renalase concentration in patients with hypertension compared to patients with normal blood pressure values is secondary to the above-mentioned relationships. Similarly, only in univariate analysis was it observed that in the group with fasting hyperglycemia the blood renalase concentration significantly more often was lower than the first quartile. The lack of significance of the above-mentioned relationship in the multivariate analysis again indicates the secondary nature of this relationship.

### 3.2. Obesity

The few studies to date on the association of renalase with obesity have described renalase as an adipokine produced by both white and brown adipose tissue. Based on animal studies, Ramanjaneya et al. tentatively linked renalase functionality to the regulation of thermogenesis, metabolism, and brown adipose tissue development, which could provide a basis for future treatment of obesity-related cardiometabolic complications [32].

In addition, Tokinoya et al. in a study on mice, linked the reduction of excess body weight to increased renalase gene expression in the kidneys and skeletal muscle [33].

The Fang et al. study on the effect of the gut microbiota on the development of obesity described the association of renalase gene knockout in mice on a normal diet with the presence of a high abundance of Firmicutes bacteria, suggesting that renalase gene knockout promotes the development of obesity or diabetes through changes in the proportions of Firmicutes and Bacteroidetes. The results of their study strongly suggest that deletion of the gene for renalase affects the composition of the microbiota and the abundance of certain bacteria in mice, and although it is pointed out that further studies are needed to describe the mechanisms in human models, it can be assumed that mouse models provide a good tool to study the pathogenesis and treatment of metabolic diseases, as they show genetic similarity to humans [34].

In a human study, Martynowicz et al. described that blood renalase concentration was reversely correlated with BMI in a group of 87 subjects [35]. Similarly, Rybi-Szumińska et al. described a statistically significant negative correlation between urinary renalase excretion levels and body mass index Z-score (r = −0.22, *p* < 0.05) in a healthy pediatric population [36].

Our research confirms the relationship between obesity and lower blood renalase concentration. At the same time, it indicates, which has not been indicated in previous studies, that this relationship is independent of other cardiovascular risk factors.

### 3.3. Smoking

Few scientific publications to date explicitly address the relationship between cigarette smoking and blood renalase concentration. Most of the studies to date focus on smoking as a factor in the development of cardiovascular disease (e.g., coronary artery disease) and the association of the latter with renalase concentration (e.g., research by Safdar et al. and Wang et al. [30,37]), rather than the direct effect of tobacco consumption on renalase concentration. One review reports on the mechanisms of regulatory promoter and transcription factors in relation to the renalase gene in which it was found that renalase promoter activity was augmented by nicotine. According to the data in this publication, individuals exposed to tobacco smoke present higher renalase concentrations than individuals without significant exposure to this harmful agent [38]. 

Our study is the first to show a clear negative relationship between smoking and blood renalase concentration, a relationship independent of the co-occurrence of other cardiovascular risk factors.

### 3.4. Physical Activity

The results of animal studies conducted to date on the effect of exercise on blood renalase concentration are partly discrepant. According to Tokinoya et al., moderate-intensity exercise performed for 60 min causes an increase in both blood renalase concentration and its expression in the skeletal muscle [39]. In another study, Tokinoya et al. reported that in addition to an increase in renalase gene expression in the muscle due to physical activity, there is an opposite effect in other tissues (kidneys, heart, and liver). According to the authors, this increase may be related to exercise-induced oxidative stress [39].

Czarkowska-Paczek et al. obtained more inhomogeneous results. No significant changes were observed either in the expression of the gene for renalase or in the concentration of renalase itself (both in the blood and in red muscle cells), both after acute and prolonged exercise. Moreover, in white muscle, gene expression and concentration of its product decreased after acute exercise in untrained rats. At the same time, renalase concentration remained unchanged in the white muscle fibres of trained rats, despite a decrease in gene expression. Based on the above, the authors concluded that exercise only affected renalase expression in white muscle fibres, which are not mainly recruited during exercise, concluding by stating that exercise differentially regulates renalase gene expression [40].

The results of our study constitute another voice in the above discussion. They indicate that there is an independent proportional relationship between physical activity and blood renalase concentration. Moreover, and importantly, they confirm the above relationship in studies conducted in the population, and not only in animal models.

### 3.5. Diet

The effect of certain elements of the diet on renalase concentration has already been described. In a study on rats conducted by Wang et al., it was shown that dietary salt intake caused a significant decrease in renalase expression in the kidneys. Furthermore, it was observed that the group with a high-salt diet presented increased systolic blood pressure and proteinuria with respect to the normal-salt diet group [41].

In human studies, by selecting 720 participants from the HyperPATH consortium program and genotyping with the use of a multiethnic genotyping array of their renalase (*RNLS*) gene, the research group of Heydarpour et al. determined that certain genetic variants of the *RNLS* gene associated with salt sensitivity of blood pressure and responded to dietary salt intervention [42]. Many other studies also confirm a decrease in blood renalase concentration caused by the influence of a high-salt diet [43,44,45].

In our study, there was no relationship between the quality of the diet assessed according to the criteria of the AHA Life 7 scale and the blood renalase concentration.

### 3.6. Arterial Hypertension

The association between blood renalase concentration, hypertension, and cardiovascular dysfunction was documented in experimental animal models and in several human studies [46]. The study by Zhao et al., which was one of the first studies in humans on the effects of renalase on cardiovascular health, described that two (rs2576178 A > G and rs2296545 C > G) of the several studied single-nucleotide polymorphisms (SNP) of the RNLS gene (the gene responsible for encoding the renalase protein) were associated with the occurrence of hypertension in northern Han Chinese [47]. Another large study conducted on the Chinese population on renalase gene polymorphisms was undertaken by Wang et al. (Baoji Salt-Sensitive Study) [37]. In a cohort study over a 14-year follow-up of 514 participants, they observed that, over the observed time period, the presence of certain renalase single-nucleotide polymorphisms (SNP) influenced the increase in blood pressure in the study population: SNP rs7922058 was associated with a change in systolic blood pressure; rs10887800, rs796945, rs1935582, rs2296545, and rs2576178 were associated with a change in diastolic blood pressure; rs1935582 and rs2576178 were associated with a change in mean arterial pressure. In addition, a group of 2392 individuals (participants from the Hanzhong Adolescent Hypertension Study cohort) were followed up for the association of renalase concentration and blood pressure. As a result, a linear correlation between serum renalase concentration and blood pressure was observed in this study. In hypertensive patients, serum renalase concentrations were higher than in those with normal blood pressure values (27.2 ± 0.4 vs. 25.1 ± 0.2 μg/mL) [37]. Subsequent studies by other research groups have also focused on the polymorphism in this gene and its impact in different populations on cardiovascular disease incidence—not only hypertension, but also cardiac hypertrophy, ventricular dysfunction, poor exercise capacity, and inducible ischemia in individuals with stable coronary artery disease [48,49,50,51]. In addition, due to the frequent co-occurrence of hypertension with type 2 diabetes, studies were also conducted that show the association of the C allele of the rs2296545 SNP occurrence with hypertension in type 2 diabetes [51]. The results obtained varied according to the study population and genotyped single-nucleotide polymorphisms—such as the fact that in the Caucasian group from the Heart and Soul Study, similar results to the northern Han Chinese population mentioned above were not obtained [52]. Thus, the incidence of relationships of renalase gene single-nucleotide polymorphisms with hypertension and other cardiovascular events should be assessed by group according to ethnicity and comorbidities in a large sample size [46].

The results of our study regarding arterial hypertension partially confirm the above views. Patients with arterial hypertension were characterised by lower blood renalase concentration. However, what must be emphasised is that the multivariate analysis did not confirm the existence of a relationship between arterial hypertension and blood renalase concentration, independent of co-occurring cardiovascular risk factors.

### 3.7. Hypercholesterolemia

Among the data evaluating the relationship between renalase concentration and cholesterol concentration, there are studies evaluating this type of relationship in kidney transplant patients: renalase positively correlated with lipoprotein metabolism disorder. However, the authors point out that such observations may be related to the fact that in renal transplant patients, a sympathetic overdrive might occur: the metabolic effect of elevated sympathetic activity is an increase in plasma total cholesterol concentration, suppression of LDL receptor activity, and a decrease in HDL cholesterol levels [53].

The above explanation may be confirmed by the results of our study, in which there was no relationship between hypercholesterolemia and blood renalase concentration in patients without chronic kidney disease.

### 3.8. Hyperglycemia

There are reports on the protective function of renalase in the context of the development of hyperglycemic complications, which include diabetic nephropathy. The protective potential of increased renalase concentration in diabetic individuals, in the context of the development of renal complications, was demonstrated in a mouse study by Yin et al. [54].

With the current understanding of renalase function as a cytokine, reports on the association of single-nucleotide polymorphisms of the renalase gene with the development of type 1 diabetes appear to be relevant. In studies by Barrett et al. and Walace et al., new loci were located and confirmed, among others (a total of 48 and 18 new ones) in the renalase gene in the region of rs10509540, located on chromosome 10q23.31, whose polymorphisms may influence the development of autoimmune destruction of pancreatic beta-cells [29,55,56].

The results of our study regarding fasting hyperglycemia only partially confirm the above views. Patients with fasting hyperglycemia were characterised by a more frequent occurrence of blood renalase concentration lower than the first quartile. However, what must be emphasized is that the multivariate analysis did not confirm the existence of a relationship between fasting hyperglycemia and blood renalase concentration, independent of co-occurring cardiovascular risk factors.

### 3.9. Study Strengths and Limitations

The strength of the research conducted is that it is a study exploring the, as yet, insufficiently investigated area of the relationship between the coexistence of cardiovascular risk factors and blood renalase concentration; however, the limitations of this study cannot be overlooked. First of all, the size of the sample group is relatively small, so further research is needed, taking into account larger group sizes to confirm our conclusions and the possibility of generalising and extrapolating them for a larger population. An additional limitation of the small group is that there was only one participant in the subgroup with only one CVRF and there was no participant with seven CVRFs. Due to obtaining more accurate results and strengthening the level of evidence, it would be necessary to repeat the study with a larger number of participants in all subgroups, especially in the subgroups with one and seven CVRFs. 

In addition, the identification of some risk factors was based on patient testimonials and, therefore, not objectively verifiable. Therefore, it cannot be ruled out that some answers missed the facts because patients wanted to perform ‘better’ in the surveys. This is because in the AHA Life 7 scale, which was used to assess the level of lifestyle ‘healthiness’ of the patients studied, some parameters are based on patient history and are not verifiable by objective measurements or laboratory tests. It can therefore be assumed that the results for the level of activity, tobacco dependence, and type of parameters may be subject to error. 

It should also be emphasised that the study conducted was designed to look for a relationship between the criteria in the AHA Life 7 scale and blood renalase concentration and to select those with the greatest relationship. Accordingly, several statistical tests were conducted, each involving the possibility of statistical error. In order to verify the validity of the selected variables and strengthen the level of evidence, it is advisable to repeat the tests to verify the adequacy of the parameters already selected (i.e., the presence of obesity, being a current smoker, and lack of physical activity is linked with a higher probability of lower blood renalase concentration).

## 4. Materials and Methods

The study was conducted in accordance with the Declaration of Helsinki and approved by the Bioethics Committee of the Wroclaw Medical University (protocol code KB-39/2020).

Group size was determined using a sample size calculator. The selection conditions were as follows: population size 2.8 million (population size of the macroregion from which patients are referred to our study center), fraction size 0.5, maximum error 10%, confidence level 95%.

The studied population was composed of 96 participants. Group description is summarised in Table 5.

All patients included in the study were hospitalised in the Department of Internal Medicine, Occupational Diseases, Hypertension, and Clinical Oncology to verify suspicions, extend diagnostics, optimise therapy, or diagnose complications of hypertension, atherosclerosis, diabetes and/or sleep disorders; and voluntarily consented to participate in the study. Patients with cancer, systemic disease, chronic kidney disease, active inflammatory process, who had a problem with clearly declaring their smoking status, or patients who had undergone cardiac- or angio-surgery or myocardial infarction, stroke or acute vascular incidents were excluded from the study. 

To measure cardiovascular health, we used a scale invented by American Heart Association (AHA): AHA Life 7, according to the survey methodology proposed by the AHA. The presence and co-occurrence of the following CVD risk factors were assessed: (1) obesity, (2) active smoking, (3) lack of physical activity, (4) unhealthy diet, (5) hypercholesterolemia, (6) fasting hyperglycemia, (7) arterial hypertension. The occurrence of CVRF was a score of 0 on the AHA Life 7 scale in each category [57].

The blood pressure measurements were taken by qualified medical personnel using clinically approved upper arm blood pressure monitors during hospitalisation. All the laboratory parameters were measured during hospitalisation. In laboratory measurements, fasting glucose, total cholesterol, and renalase concentrations were determined. Blood was collected by ulnar venipuncture and kept at a stable temperature. E3109Hu ELISA kit (enzyme-linked immunosorbent assay) (Bioassay Technology Laboratory, Shanghai, China) was used to determine blood renalase concentration, in accordance with the manufacturer’s instructions. As specified by the producer, the parameters of the test used were as follows. The reference range: 1–400 ng/mL, the sensitivity of the ELISA: 0.52 ng/mL, the coefficient of intra- and inter-assay variation: <8% and <10%, respectively.

The variables related to the blood renalase concentration (quantitative variable: blood renalase concentration, and categorical variables: renalase concentration < 1st quartile, renalase concentration ≥ median, renalase concentration ≥ 3rd quartile) were compared with each CVRF and with the number CVRF.

We used Statistica 13 TIBCO Software Inc. provided by StatSoft Poland to conduct all analyses. Categorical variables are presented as absolute values and percentages. We used Chi-square test to conduct categorical analysis. The arithmetic means and standard deviations were calculated for quantitative variables. The normality of the distribution of quantitative variables was verified. For quantitative variables with a normal distribution, the *t*-test or ANOVA (one-way parametric) analysis of variance with post-hoc tests was used for further statistical analysis. For quantitative variables with a non-normal distribution, the Mann–Whitney U test or a non-parametric equivalent of the analysis of variance, the Kruskal–Wallis ANOVA, and post-hoc tests were used. Relationships between variables were assessed using correlation analysis and multivariate regression analysis. In addition, the assessment of the sensitivity and specificity was made. A result at the level of *p* < 0.05 was considered significant.

## 5. Conclusions

In conclusion, we found that patients with a higher number of cardiovascular risk factors had lower blood renalase concentrations. The cardiovascular risk factors that we found most strongly associated with lower blood renalase concentration were obesity, smoking, and lack of physical activity. A blood renalase concentration lower than 38.98 ng/mL was the most accurate predictor of having at least five cardiovascular risk factors.

## Figures and Tables

**Table 1:** Blood renalase concentration in groups distinguished based on individually analysed cardiovascular disease risk factors.

		Renalase ^b^ [ng/mL]	Renalase < 1st Quartile ^a^	Renalase ≥ Median ^a^	Renalase ≥ 3rd Quartile ^a^
Active smoking	yes	102.77 ± 185.47	18/66.7	3/11.1	3/11.1
	no	255.98 ± 217.02	6/8.7	45/65.2	21/30.4
	*p*	<0.05	<0.05	<0.05	<0.05
Obesity	yes	107.06 ± 129.48	12/40.0	8/26.7	3/10.0
	no	260.99 ± 234.61	12/18.2	40/60.6	21/31.8
	*p*	<0.05	<0.05	<0.05	<0.05
Lack of physical activity	yes	137.87 ± 172.97	11/35.5	10/32.3	4/12.9
	no	248.67 ± 230.40	13/20.0	38/58.5	20/30.8
	*p*	<0.05	ns	<0.05	ns
Unhealthy diet	yes	184.12 ± 215.29	13/35.1	15/40.5	8/21.6
	no	230.93 ± 221.06	11/18.6	33/55.9	16/27.1
	*p*	ns	ns	ns	ns
Hypercholesterolemia	yes	193.12 ± 198.85	10/23.8	21/50.0	9/21.4
	no	228.27 ± 234.01	14/25.9	27/50.0	15/27.8
	*p*	ns	ns	ns	ns
Arterial hypertension	yes	156.13 ± 176.59	13/38.2	13/38.2	6/17.6
	no	244.02 ± 233.08	11/17.7	35/56.4	18/29.0
	*p*	<0.05	<0.05	ns	ns
Fasting hyperglycemia	yes	111.67 ± 133.61	4/50.0	2/25.0	1/12.5
	no	222.09 ± 223.31	20/22.7	46/52.3	23/26.1
	*p*	ns	<0.05	ns	ns

^a^ The values represent absolute values/percentages; ^b^ values represent means ± standard deviation; ns—non-significant.

**Table 2:** Blood renalase concentration in groups distinguished based on the number of identified risk factors for cardiovascular diseases.

		Renalase ^b^ [ng/mL]	Renalase < 1st Quartile ^a^	Renalase ≥ Median ^a^	Renalase ≥ 3rd Quartile ^a^
CVRF number	0–1	284.33 ± 232.20	2/5.4	26/70.3	13/35.1
	2–3	207.97 ± 218.40	11/28.2	19/48.7	9/23.1
	≥4	90.33 ± 127.50	11/55.0	3/15.0	2/10.0
	*p*	0–1 vs. ≥4: <0.05 2–3 vs. ≥4: <0.05	0–1 vs. ≥4: <0.05	0–1 vs. ≥4: <0.05 2–3 vs. ≥4: <0.05	ns
CVRF number = 0	yes	305.47 ± 213.61	0/0.0	9/81.8	4/36.4
	no	200.91 ± 217.94	24/28.2	39/45.9	20/23.5
	*p*	ns	<0.05	<0.05	ns
CVRF number > Me (>2)	yes	92.59 ± 111.30	17/45.9	9/24.3	3/8.1
	no	288.34 ± 236.18	7/11.9	39/66.1	21/35.6
	*p*	<0.05	<0.05	<0.05	<0.05

^a^ The values represent absolute values/percentages; ^b^ values represent means ± standard deviation; CVRF—cardiovascular risk factor; ns—non-significant.

**Table 3:** Results of estimation for model obtained in progressive stepwise multivariable analysis of regression.

Model for: Renalase [ng/mL]				
	Regression Coefficient	SEM of Rc	*p*	*p* of the Model
Intercept	333.195	34.938	<0.001	<0.001
Obesity	−121.748	44.582	<0.001	
Active smoking	−118.151	46.217	<0.05	
Lack of physical activity	−87.029	43.570	<0.05	

SEM of Rc—standard error of the mean of regression coefficient.

**Table 4:** The sensitivity and specificity of blood renalase concentration as a predictor of the number of CVRFs.

	CVRF ≥ 1	CVRF ≥ 2	CVRF ≥ 3	CVRF ≥ 4	CVRF ≥ 5
Blood renalase concentration as predictor of number of CVRFs [ng/mL]	<93.33	<89.41	<89.41	<75.59	<38.98
Sensitivity	0.846	0.703	0.661	0.658	0.874 *
Specificity	0.578	0.644	0.784	0.800 **	0.556
Accuracy	0.615	0.667	0.708	0.688	0.844 ***
Positive predictive values	0.239	0.553	0.830	0.926	0.950
Negative predictive values	0.960	0.776	0.592	0.381	0.313
Likelihood ratios positive	2.007	1.974	3.057	3.289	1.966
Likelihood ratios negative	0.266	0.462	0.432	0.428	0.228

* Highest prediction sensitivity; ** highest prediction specificity; *** highest prediction accuracy; CVRF—cardiovascular risk factor.

**Table 5:** Basic clinical parameters in the whole study group.

	Whole Study Group (n = 96)	Men (M) (n = 47)	Women (W) (n = 49)	P M vs. W
Age ^b^ [years]	48.51 ± 14.73	50.64 ± 14.12	46.47 ± 15.15	ns
Male gender ^a^	47/49.0	-	-	-
Female gender ^a^	49/51.0	-	-	-
BMI ^b^ [kg/m^2^]	27.82 ± 4.78	27.92 ± 3.77	27.71 ± 5.61	ns
Obesity ^a^	33/34.4	17/36.2	13/26.5	ns
Systolic blood pressure ^b^ [mmHg]	136.77 ± 19.65	139.26 ± 21.57	134.39 ± 17.52	ns
Diastolic blood pressure ^b^ [mmHg]	87.24 ± 11.17	88.09 ± 11.96	86.43 ± 10.41	ns
Arterial hypertension ^a^	34/35.4	20/42.5	14/28.6	ns
Total cholesterol ^b^ [mg/dL]	217.48 ± 39.17	225.49 ± 38.61	210.57 ± 37.94	ns
Hypercholesterolemia ^a^	42/43.7	24/51.1	18/36.7	ns
Glucose ^b^ [mg/dL]	116.75 ± 41.18	114.03 ± 38.71	118.52 ± 40.92	ns
Diabetes mellitus ^a^	8/8.3	4/8.5	4/8.2	ns
Smoking ^a^	27/28.1	15/31.9	12/24.5	ns
CVRF number ^b^	2.19 ± 1.42	2.43 ± 1.44	1.96 ± 1.37	ns
Renalase ^b^ [ng/mL]	212.89 ± 218.91	191.94 ± 193.34	232.95 ± 241.21	ns

^a^ The values represent absolute values/percentages; ^b^ values represent means ± standard deviation; BMI—body mass index; CVRF—cardiovascular risk factor; ns—non-significant.

## Data Availability

The data presented in this study are available on request from the corresponding author. The data are not publicly available due to privacy.
